# Liquid scintillation counters calibration stability over long timescales

**DOI:** 10.1007/s10967-017-5388-6

**Published:** 2017-08-09

**Authors:** Pawel Gaca, Phillip E. Warwick, Ian W. Croudace

**Affiliations:** University of Southampton, Ocean & Earth Science, National Oceanography Centre, European Way, Southampton, SO14 3ZH UK

**Keywords:** LSC calibration, Radionuclide measurement, Radioactivity analysis, 1220 Quantulus

## Abstract

Liquid scintillation spectrometry is widely used for the analysis of alpha and beta emitting radionuclides. Robust calibration of liquid scintillation (LS) spectrometers is fundamental to accurate LS measurement but at the same time is time consuming and costly, particularly if a wide range of radionuclides are analysed by the laboratory. The frequency of the calibration varies in different laboratories and is based on many practical and operational factors. This work summarizes the observations regarding variations in 1220 Quantulus spectrometers efficiency calibrations performed annually using various radionuclides: ^3^H ^63^Ni, ^55^Fe, ^36^Cl, ^45^Ca, ^147^Pm, ^241^Pu, ^99^Tc for a period of 9 years and discusses the implication to calibration frequency.

## Introduction

Liquid scintillation (LS) spectrometry has been a very important tool in radiation measurement field since mid-twentieth century when first attempts to measure ^14^C by this method were made [1]. The technique has developed over years and nowadays the liquid scintillation spectrometers can be found in many institutions related to a wide area of applications: from nuclear industry and safety, through radiopharmaceutical industry to a number of research-related organizations (universities, research institutes). Modern LS counters offer the possibility of both qualitative and quantitative analysis of alpha and beta emitters with high precision and accuracy, offering at the same time low detection limits. As many other instrumental techniques, liquid scintillation spectrometry requires robust and reliable calibration to accurately measure radiation [2, 3]. However, the detection efficiency of the counting equipment will vary depending on a range of factors including radionuclide emission and associated energy, sample composition, scintillator and vial type. All these factors must be accurately quantified and corrected for during routine measurement. In addition, the detection efficiency will vary with time due to deterioration of instrument optics and periodic recalibration is therefore required. In order to address these issues the most commonly used approach is to calibrate the LS spectrometers using a set of standards of known activity and a varying amount of quench.

Full calibration of a liquid scintillation counter is time consuming, particularly if a wide range of radionuclides are analysed by the laboratory. It can also be expensive if frequent purchase of new certified standard is required e.g., for radionuclides with short half-live in a range of days or weeks. Use of high energy beta emitters (e.g. ^90^Sr/^90^Y or ^32^P) may also carry an additional radiological risk.

The frequency at which full calibrations are performed will depend on the laboratory and is based on a range of practical and operational factors. However, to inform this choice, it is useful to understand the long term stability of liquid scintillation counters and the rate of degradation of the system. In addition, knowledge of the long term stability of the LS calibration can be used in assessing method uncertainties associated with calibration. This work presents the summary of the Quantulus LSC calibrations performed annually using various radionuclides: ^63^Ni, ^55^Fe, ^36^Cl, ^45^Ca, ^147^Pm, ^241^Pu, ^99^Tc for a period of 10 years and assesses the long term stability of the instruments.

## Experimental

A set of calibration equations for three different 1220 Quantulus counters (Quantulus 1, Quantulus 2 and Quantulus 3), obtained between years 2007 and 2016, was used. Radionuclides considered are presented in Table 1. Counting efficiency calibrations for all the radionuclides were prepared using certified standard solutions traceable to National Physical Laboratory (UK), National Institute for Standard and Technology (USA) or Physikalish-Technische Bunsesanstaldt (Germany). The different quench level for any given set of calibration standards was achieved by the addition of varying amounts of a quenching agent (e.g. nitromethane) or by using different sample solution to scintillation cocktail ratios. As the quench level estimation method the SQPE (external standard quench parameter) approach was used [4]. This method is based on the comparison of two spectra: first one is obtained for the sample exposed to the gamma radiation from the external source, second one is obtained for the sample alone. Both spectra are collected for the same amount of time and the non-exposed spectrum is subtracted from the exposed one. The channel number where 99% of the net spectrum counts lay below is used as the measure of quench in the analysed sample. The details on calibration standards preparation were combined in Table 2. The deviations in counting efficiencies were checked for selected SQPE values representing varying degree of quench; from heavily quenched samples (SQPE 1), through a mid-level (SQPE 2) to relatively weakly affected ones (SQPE 3). The SQPE values used for the comparison purposes were fixed for any given radionuclide and the calibration date and are presented in Table 3 together with a typical calibration curve fit uncertainty (2*σ*) observed in the considered timescale. Each calibration curve fit (2nd degree polynomial) was used to calculate counting efficiencies for each radionuclide at different SQPE value. The obtained values were plotted against calibrtation date.

**Table 1:** Radionuclides used for counting efficiency stability check

Isotope	*T*_1/2_	*β*_max_ (keV)	Number of calibration standard stock solutions used between 2007 and 2016
^3^H	12.33a	18.571	6, traceable to different original standard solutions
^241^Pu	14.4a	20.81	2, traceable to different original standard solutions
^55^Fe	2.735a	EC	4, traceable to different original standard solutions
^63^Ni	99a	65.87	2, traceable to different original standard solutions
^147^Pm	2.622a	224.1	2, traceable to single original standard solution
^45^Ca	162.7d	256.9	4, traceable to different original standard solutions
^99^Tc	211,300a	293.6	1, traceable to single original standard solution
^36^Cl	302,000a	709.6	3, traceable to single original standard solution

a: years, d: days

**Table 2:** Calibration standards preparation details

Nuclide	Final source matrix	Quenching agent	Volume range (ml)	Scintillation cocktail	Total final volume (ml)
^3^H	H_2_O or 0.1 M HNO_3_	0.1 M HNO_3_	3–10	Gold Star™	20
^36^Cl	1:1 0.88 S.G. NH_4_OH & H_2_O	1:1 0.88 S.G. NH_4_OH & H_2_O	1–5	ProFlowP+™	20
^45^Ca	3 M HCl	3 M HCl	0–8	Gold Star™	20
^55^Fe	2 M H_3_PO_4_	2 M H_3_PO_4_	1–8	Ultima Gold AB™	18
^63^Ni	1.2 M HCl	1.2 M HCl	1–8	Gold Star™	18
^99^Tc	5% TOA/xylene	Nitromethane	5 ml TOA/xylene and 0–0.1 ml nitromethane	Gold Star™	20
^147^Pm	1.2 M HCl	1.2 M HCl	0–5	Gold Star™	20
^241^Pu	TOPO/toluene	Nitromethane	4 ml of TOPO/toluene and 0–0.08 ml nitromethane	Gold Star™	10

**Table 3:** Quench parameters (SQPE) values used for calibration stability comparison and typical calibration curve fit uncertainty (2*σ*)

Isotope	Quench level	Quantulus 1	Quantulus 2	Quantulus 3	Typical calibration curve fit uncertainty (2*σ*)
^3^H	SQPE 1	730	710	730	2%
	SQPE 2	770	740	770	
	SQPE 3	800	780	800	
^241^Pu	SQPE 1	600	600	620	2%
	SQPE 2	700	650	710	
	SQPE 3	820	810	900	
^55^Fe	SQPE 1	800	770	820	3%
	SQPE 2	825	810	870	
	SQPE 3	860	835	920	
^63^Ni	SQPE 1	740	720	810	4%
	SQPE 2	800	770	855	
	SQPE 3	850	815	900	
^147^Pm	SQPE 1	750	740	830	2%
	SQPE 2	790	770	880	
	SQPE 3	830	810	920	
^45^Ca	SQPE 1	710	700	780	3%
	SQPE 2	770	750	830	
	SQPE 3	830	810	880	
^99^Tc	SQPE 1	670	630	715	3%
	SQPE 2	730	730	810	
	SQPE 3	800	810	890	
^36^Cl	SQPE 1	730	700	795	3%
	SQPE 2	770	730	825	
	SQPE 3	800	750	855	

## Results and discussion

The obtained results for variation in counting efficiency between years 2007 and 2016 were plotted for each radionuclide, spectrometer and quench level. A linear trendline was fitted to each set of data—the slope parameter of the obtained fit equation represents a rate of annual change in the absolute counting efficiency. For each individual spectrometer and quench level an average annual absolute counting efficiency change was calculated. These numbers were further averaged to produce a single value representing an annual change in absolute counting efficiency for all three spectrometers. The summary of the obtained results for ^3^H, ^241^Pu, ^55^Fe, ^63^Ni, ^147^Pm, ^45^Ca, ^99^Tc and ^36^Cl is presented in Tables 4, 5, 6, 7, 8, 9, 10, 11 and 12, respectively. For ^147^Pm two separate tables (Tables 8, 9) are presented to reflect the change in counting window modification from 1–600 channels to 301–650 channels. The counting window modification was introduced to allow the elimination of potential of ^151^Sm (*β*_max_ = 76.3 keV) interference with ^147^Pm measurement as both elements show similar chemical properties and the complete isolation of the two radionuclides is challenging.

**Table 4:** Annual counting efficiency change for ^3^H

Spectrometer	Quench level	linear fit slope	*R* ^2^ (linear fit)	Δ absolute counting efficiency (max to min value) (%)	Average annual absolute counting efficiency change (%)	Average annual absolute counting efficiency change for all counters (%)
Quantulus 1	SQPE 1	−0.5675	0.4910	6.96	−0.53	−0.54
	SQPE 2	−0.5672	0.9226	4.69		
	SQPE 3	−0.4681	0.9158	3.85		
Quantulus 2	SQPE 1	−0.5479	0.4343	9.19	−0.49	
	SQPE 2	−0.5133	0.4310	8.71		
	SQPE 3	−0.4088	0.4246	6.75		
Quantulus 3	SQPE 1	−0.7189	0.9078	7.33	−0.58	
	SQPE 2	−0.6451	0.9388	6.37		
	SQPE 3	−0.3835	0.1742	7.82

**Table 5:** Annual counting efficiency change for ^241^Pu

Spectrometer	Quench level	linear fit slope	*R* ^2^ (linear fit)	Δ absolute counting efficiency (max to min value) (%)	Average annual absolute counting efficiency change (%)	Average annual absolute counting efficiency change for all counters (%)
Quantulus 1	SQPE 1	−0.9009	0.6190	9.73	−0.56	−0.59
	SQPE 2	−0.2146	0.8074	2.27		
	SQPE 3	N/A	N/A	6.51		
Quantulus 2	SQPE 1	−0.7105	0.5548	11.71	−0.52	
	SQPE 2	−0.3283	0.7322	4.52		
	SQPE 3	N/A	N/A	5.09		
Quantulus 3	SQPE 1	−1.0875	0.6259	7.94	−0.68	
	SQPE 2	−0.2811	0.3808	3.71		
	SQPE 3	N/A	N/A	4.36

**Table 6:** Annual counting efficiency change for ^55^Fe

Spectrometer	Quench level	linear fit slope	*R* ^2^ (linear fit)	Δ absolute counting efficiency (max to min value) (%)	Average annual absolute counting efficiency change (%)	Average annual absolute counting efficiency change for all counters (%)
Quantulus 1	SQPE 1	−1.7755	0.5287	21.47	−1.22	−0.90
	SQPE 2	−1.4054	0.5902	15.39		
	SQPE 3	−0.4849	0.1185	13.10		
Quantulus 2	SQPE 1	−1.3900	0.5999	14.79	−0.73	
	SQPE 2	−1.1338	0.4634	13.11		
	SQPE 3	0.3426	0.113	12.01		
Quantulus 3	SQPE 1	−1.5344	0.5106	19.36	−0.75	
	SQPE 2	−0.9316	0.4654	16.40		
	SQPE 3	0.2017	0.0462	10.37

**Table 7:** Annual counting efficiency change for ^63^Ni

Spectrometer	Quench level	linear fit slope	*R* ^2^ (linear fit)	Δ absolute counting efficiency (max to min value) (%)	Average annual absolute counting efficiency change (%)	Average annual absolute counting efficiency change for all counters (%)
Quantulus 1	SQPE 1	−0.1786	0.0207	15.04	−0.11	−0.10
	SQPE 2	−0.0107	0.0019	3.09		
	SQPE 3	−0.1519	0.0618	5.71		
Quantulus 2	SQPE 1	−0.0303	0.0028	5.17	−0.03	
	SQPE 2	0.1451	0.2711	2.17		
	SQPE 3	−0.2101	0.1111	5.04		
Quantulus 3	SQPE 1	−0.4187	0.1699	10.55	−0.16	
	SQPE 2	0.0245	0.0245	2.68		
	SQPE 3	−0.0992	0.0088	11.12

**Table 8:** Annual counting efficiency change for ^147^Pm before counting window modification

Spectrometer	Quench level	linear fit slope	*R* ^2^ (linear fit)	Δ absolute counting efficiency (max to min value) (%)	Average annual absolute counting efficiency change (%)	Average annual absolute counting efficiency change for all counters (%)
Quantulus 1	SQPE 1	0.5167	0.4891	4.00	0.38	0.33
	SQPE 2	0.4341	0.6111	3.43		
	SQPE 3	0.192	0.0471	5.82		
Quantulus 2	SQPE 1	0.3487	0.5230	2.47	0.37	
	SQPE 2	0.2680	0.6402	1.79		
	SQPE 3	0.4788	0.8407	1.70		
Quantulus 3	SQPE 1	0.2779	0.1077	5.53	0.25	
	SQPE 2	0.3205	0.7330	2.02		
	SQPE 3	0.1531	0.1559	2.49

**Table 9:** Annual counting efficiency change for ^147^Pm after counting window modification

Spectrometer	Quench level	linear fit slope	*R* ^2^ (linear fit)	Δ absolute counting efficiency (max to min value) (%)	Average annual absolute counting efficiency change (%)	Average annual absolute counting efficiency change for all counters (%)
Quantulus 1	SQPE 1	0.4550	0.9149	0.91	0.41	0.29
	SQPE 2	0.0622	0.0322	0.65		
	SQPE 3	0.7202	0.9492	1.44		
Quantulus 2	SQPE 1	0.2118	0.9184	0.42	0.25	
	SQPE 2	0.3001	0.9295	0.60		
	SQPE 3	0.2423	0.1510	1.24		
Quantulus 3	SQPE 1	0.1802	0.0290	1.99	0.21	
	SQPE 2	0.1691	0.9725	0.34		
	SQPE 3	0.2934	0.6804	0.64

**Table 10:** Annual counting efficiency change for ^45^Ca

Spectrometer	Quench level	linear fit slope	*R* ^2^ (linear fit)	Δ absolute counting efficiency (max to min value) (%)	Average annual absolute counting efficiency change (%)	Average annual absolute counting efficiency change for all counters (%)
Quantulus 1	SQPE 1	−0.3464	0.0987	11.35	−0.28	−0.40
	SQPE 2	−0.3405	0.4276	5.26		
	SQPE 3	−0.1662	0.1317	4.88		
Quantulus 2	SQPE 1	−0.6694	0.1470	18.48	−0.43	
	SQPE 2	−0.3990	0.7487	4.79		
	SQPE 3	−0.2225	0.1546	5.87		
Quantulus 3	SQPE 1	−0.6753	−0.6753	10.56	−0.47	
	SQPE 2	−0.2899	0.6888	3.47		
	SQPE 3	−0.4559	0.6347	5.12

**Table 11:** Annual counting efficiency change for ^99^Tc

Spectrometer	Quench level	linear fit slope	*R* ^2^ (linear fit)	Δ absolute counting efficiency (max to min value) (%)	Average annual absolute counting efficiency change (%)	Average annual absolute counting efficiency change for all counters (%)
Quantulus 1	SQPE 1	0.2948	0.4976	3.77	0.15	0.22
	SQPE 2	0.1404	0.1116	4.16		
	SQPE 3	0.0063	0.00005	9.05		
Quantulus 2	SQPE 1	0.3528	0.8262	3.48	0.25	
	SQPE 2	0.2314	0.7089	2.30		
	SQPE 3	0.1764	0.0098	16.14		
Quantulus 3	SQPE 1	0.2715	0.6590	2.61	0.25	
	SQPE 2	0.3471	0.7494	3.43		
	SQPE 3	0.1397	0.0037	22.58

**Table 12:** Annual counting efficiency change for ^36^Cl

Spectrometer	Quench level	linear fit slope	*R* ^2^ (linear fit)	Δ absolute counting efficiency (max to min value) (%)	Average annual absolute counting efficiency change (%)	Average annual absolute counting efficiency change for all counters (%)
Quantulus 1	SQPE 1	1.0105	0.6114	12.37	0.42	0.31
	SQPE 2	0.2546	0.3872	4.19		
	SQPE 3	−0.0005	0.0000009	5.93		
Quantulus 2	SQPE 1	0.1411	0.1114	4.39	0.15	
	SQPE 2	0.0217	0.0011	7.19		
	SQPE 3	0.2985	0.0178	22.85		
Quantulus 3	SQPE 1	0.5402	0.5134	6.46	0.35	
	SQPE 2	0.2441	0.1848	5.14		
	SQPE 3	0.2545	0.1276	6.66		

**Fig. 1:**
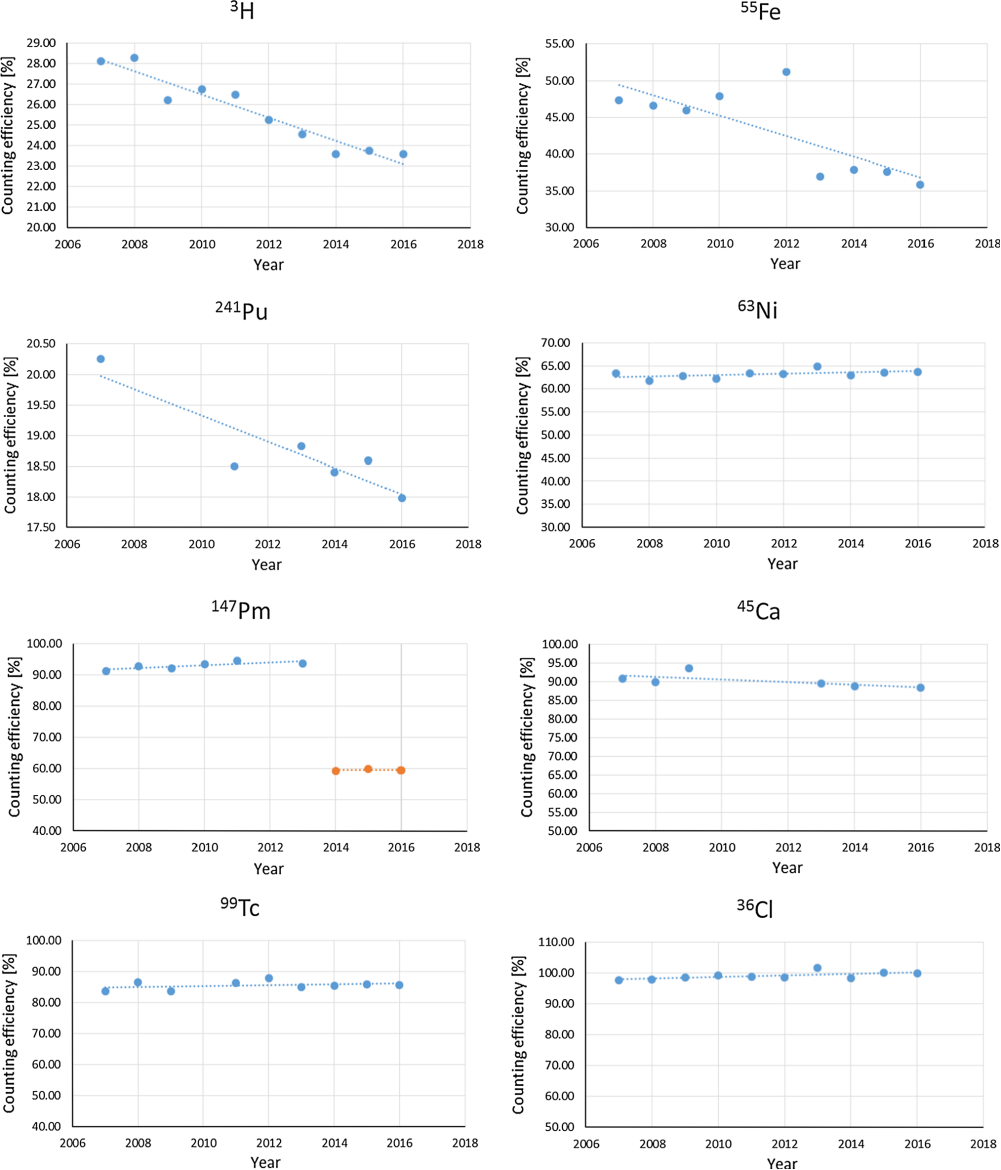
Counting efficiency change plots for Quantulus 1 at medium quench level (SQPE 2). Gaps in data series are caused by lack of data (no calibration because of standard unavailability). Step change in ^147^Pm in 2014 counting efficiency is the result of modification to the counting window selected for the analysis

Plots for the observed counting efficiency change in time for Quantulus 1 and medium quench level, typical for most of the analysed samples, are presented in Fig. 1.

Absolute counting efficiency for low energy beta emitters (^3^H, ^241^Pu) and ^55^Fe drops by approximately 0.5–1% every year. Similar observation for ^3^H was observed previously [2]. Such relatively high degradation rate of the counting efficiency may be explained by slow deterioration of the counter optics caused by the slow release of vapours (both acidic and basic) from the counted sources over time and progressive dust particles accumulation. Since the light originating from low-energy beta emitters is sensitive to any disturbance in the optics of the instrument due to its lower intensity, taking a greater care on the instruments condition (e.g., annual cleanup) should result in reduced counting efficiency deterioration. A step change in counting efficiency visible for ^55^Fe in year 2012 was caused by the use of a new standard solution. This solution was used for the calibration purposes only in year 2012 and was replaced in the following years by other ^55^Fe standard solutions.

Radionuclides emitting more energetic beta radiation show much greater counting efficiency stability over long time periods which was previously observed for ^14^C [5]. During current checks a slight positive change can be observed for ^147^Pm, ^99^Tc and ^36^Cl. As an increase of the spectrometer performance in time is very unlikely, the effect can most probably be explained by the slow evaporative loss and associated concentration of the stock solutions used to prepare the calibration standards. Typical initial mass of the freshly prepared stock solution is approx. 30 g. Activity concentration change of 0.2% every year (as suggested by ^99^Tc counting efficiency increase) leads to activity change of ca. 2% over the study period of nine years. This corresponds to approximately mass change of 0.6 g over nine years or, on average, 0.07 g/year. Because the standard bottle is usually opened many times every year to produce calibration standards, test sources, quality control solutions, etc., it is likely that the standard solution evaporation is the cause of the observed positive counting efficiency change for some radionuclides. Similar analysis could be done for ^147^Pm and ^36^Cl standards where the positive counting efficiency change trend of approximately 0.3%/year can be observed, leading to an annual average solution mass losses of 0.09 g.

Such a hypothesis may be further strengthened by the fact that all the calibration standards used for these three radionuclides can be traced back to a single original stock solution bottle stored for several years in a PFA bottle at ambient temperature. Further, the analysis of ^45^Ca behavior shows a decrease in counting efficiency similar to low-energy beta emitters, ca. 0.4% every year. In this case the calibration standards used to calibrate the spectrometers can be traced back to different original certified standards—mainly because of a short ^45^Ca half-live preventing the use of one stock solution over a long timescale.

Taking into account the fact that for the low-energy beta emitters, where counting efficiency is typically 20–30%, the absolute counting efficiency change by 1% every year translates to a relative counting efficiency change between 3 and 5%, care must be taken to observe the trend and update the counting efficiency accordingly on more frequent basis or to increase the total method uncertainty to allow for such a rapid change [6]. For high energy beta emitters, where typical counting efficiencies are in the range of 80–100% the effect is much less significant and corresponds to relative counting efficiency change at a level of 1%. Of course such changes, however small, should also be taken into account while constructing the expanded uncertainty budget.

One of possible conclusions from the calibration stability comparison for different radionuclides is that for an instrument with “undisturbed” (not cleaned or heavily serviced) optics/photomultiplier tubes windows, the counting efficiency drops at a steady rate between 0.5 and 1% every year. This degradation rate can be observed over a fairly broad range of beta emission energies of the considered radionuclides. The more stable counting efficiency behaviour for high-energy beta emitters may be a genuine stability as well as an apparent effect caused by the dropping counter efficiency balanced by the slow activity concentration increase of the primary standard solutions used to prepare the calibration standards.

## Conclusions

The degree of the observed changes in counting efficiency is similar for all three instruments used in this comparison which suggests that the degradation of the electronic and/or optical components of each system takes place at similar pace. These similarities seem to be linked to the external environment (instrument location) and chemical form of the samples being counted as the most important factors influencing the long-term stability of the counters. The reliability of the radioactive standard solutions over long timescale seems to be another factor which could influence the calibration of the spectrometers, as evaporation, adsorption on the container walls or any other not yet identified process may lead to alteration of the specific activity of a standard solution which in turn causes errors in any calculations which are based on the activity measurements of such solutions.

Depending on particular analytical needs, it is possible to extend the calibration periods for medium and high-energy beta emitters. For low-energy beta emitters the calibration frequency should depend on the observation of the trend in counting efficiency change. In both cases the rate of counting efficiency change should be built into the overall measurement uncertainty budget.
